# Impact of Empowering Leadership on Antimicrobial Stewardship: A Single Center Study in a Neonatal and Pediatric Intensive Care Unit and a Literature Review

**DOI:** 10.3389/fped.2018.00294

**Published:** 2018-10-12

**Authors:** Karin E. Steinmann, Dirk Lehnick, Michael Buettcher, Katharina Schwendener-Scholl, Karin Daetwyler, Matteo Fontana, Davide Morgillo, Katja Ganassi, Kathrin O'Neill, Petra Genet, Susanne Burth, Patrizia Savoia, Ulrich Terheggen, Christoph Berger, Martin Stocker

**Affiliations:** ^1^Department of Pediatrics, Children's Hospital Lucerne, Lucerne, Switzerland; ^2^Department of Health Sciences and Health Policy, Biostatistics and Methodology, University Lucerne, Lucerne, Switzerland; ^3^Infectious Diseases Unit, Children's Hospital Lucerne, Lucerne, Switzerland; ^4^Pediatric and Neonatal Intensive Care Unit, Children's Hospital Lucerne, Lucerne, Switzerland; ^5^Department of Infectious Diseases and Hospital Epidemiology and Children's Research Center, University Children's Hospital Zurich, Zurich, Switzerland

**Keywords:** leadership, empowerment, shared leadership, distributed leadership, antimicrobial stewardship, intensive care unit, pediatrics

## Abstract

**Background:** Antimicrobial stewardship (AMS) is an important strategy of quality improvement for every hospital. Leadership is an important factor for implementation of quality improvement and AMS programs. Recent publications show successful AMS programs in children's hospitals, but successful implementation is often difficult to achieve and literature of AMS in neonatal and pediatric intensive care units (NICU/PICU) is scarce. Lack of resources and prescriber opposition are reported barriers. A leadership style focusing on empowering frontline staff to take responsibility is one approach to implement changes in health care institutions.

**Aim:** Literature review regarding empowering leadership and AMS in health care and assessment of the impact of such a leadership style on AMS in a NICU/PICU over 3 years.

**Methods:** Assessment of the impact of a leadership change September 1, 2015 from control-driven to an empowering leadership style on antibiotic use and hospital acquired infections. Prospective analysis and annual comparison of antibiotic use, rate of suspected and confirmed ventilator-associated pneumonia (VAP) and central-line associated blood stream infection (CLABSI) including antibiotic use overall, antibiotic therapy for culture-negative and culture-proven infections including correct initial choice and streamlining of antibiotics in the NICU/PICU of the Children's Hospital of Lucerne between January 1, 2015 and December 31, 2017.

**Results:** Five articles were included in the literature review. All five studies concluded that an empowering leadership style may lead to a higher engagement of physicians. Three out of five studies reported improved AMS as reduced rate in hospital-acquired infections and improved prevention of MRSA infections. From 2015 to 2017, antibiotic days overall and antibiotic days for culture-negative situations (suspected infections and prophylaxis) per 1000 patient days declined significantly from 474.1 to 403.9 and from 418.2 to 309.4 days, respectively. Similar, the use of meropenem and vancomycin declined significantly. Over the 3 years, suspected and proven VAP- and CLABSI-episodes decreased with no confirmed episodes in 2017.

**Conclusion:** An empowering leadership style which focuses on enabling frontline physicians to take direct responsibilities for their patients may be a successful strategy of antimicrobial stewardship allowing to overcome reported barriers of AMS implementation.

## Introduction

Quality improvement in healthcare has become one of the most discussed topics of the Twenty-first century and has real potential to enhance patients' outcome. The emergence of outcome measurement and quality improvement in the neonatal intensive care units (NICU) showed a profound effect on improving outcomes for premature neonates within the last decade (1). Antimicrobial stewardship (AMS) is an important strategy of quality improvement. The Infectious Disease Society of America describe the primary goal of AMS as optimizing clinical outcomes while minimizing unintended consequences of antimicrobial use, including toxicity, the selection of pathogenic organisms, and the emergence of resistance (2, 3). A recent published systematic review regarding AMS in children's hospitals showed significant reduction of overall and/or selected antibiotic use in general pediatric wards, whereas only limited information is available on effective components of successful AMS programs in pediatric and neonatal intensive care units (PICU/NICU) (4).

Antibiotics are among the most used medications in PICUs and NICUs, often prescribed for culture-negative situations (5–8). Especially neonates and young infants often present with unspecific clinical and laboratory signs of infections and empirical antibiotic therapy has to be started (9, 10). This is mandatory, as delayed antimicrobial therapy causes increased morbidity and mortality of truly infected children (9, 11). On the other hand, inappropriate prescription of antibiotics may have serious short-term consequences for children who eventually do not suffer from infection: drug-related adverse events, longer hospital stay, increased risk of fungal infections, rate of necrotizing enterocolitis (NEC), healthcare costs and mortality (12). In addition, alongside the known long-term consequences of inappropriate antibiotic use such as antimicrobial resistance (13–16), there is a growing body of evidence on the impact on the human microbiome with potential consequences for future health, especially when antibiotics are prescribed in the first few years of life (10, 12, 14, 17, 18). Therefore, appropriate pediatric AMS programs, particularly in NICUs and PICUs are urgently needed (4, 9, 19). Successful implementation of AMS is often a challenge due to the need of a high amount of staff resources (20), specific staff education, the implementation of an inter-professional AMS team, and lack of financial support from hospital administrations (4, 16, 19, 21, 22). Prescriber opposition due to the control-driven aspect of many AMS components may be an additional barrier for efficient implementation (21). The need for a strong leadership in these programs is often mentioned but rarely defined. A leadership style focusing on empowering frontline physicians to take over individual responsibility in the application of AMS is a potential strategy to improve the outcome of children receiving antibiotics or interventions, even when the respective staff and financial resources for a comprehensive AMS program are not available.

The aim of this study was first to perform a literature review regarding empowering leadership style for physicians with a specific focus on AMS. Secondly, we wanted to analyze the impact of a leadership style empowering frontline physicians to take decisions situational on AMS in our PICU and NICU over the last 3 years. We hypothesized that, with this leadership style, antibiotic days overall and antibiotic days for culture-negative situations (suspected infections and prophylaxis) decreases. In addition, we hypothesized that suspected hospital-acquired infections and antibiotic days for suspected hospital-acquired infections decrease.

## Methods

### Literature review

We conducted a literature review searching the database PubMed according to following key words: “leader^*^ AND antimicrobial stewardship,” “shared leadership AND physician AND patient outcome,” “distributed leadership AND health care,” “empowerment AND antimicrobial stewardship,” “empowerment AND front line AND patient outcome,” such as “patient outcome AND leadership style NOT nurse.” As we focused on analyzing leadership styles among physicians and especially with physicians as leaders and stakeholders/followers, we excluded literature regarding leadership styles in nursing or business and management journals without relation to physicians. Comments, guidelines and reviews were excluded, only quantitative and qualitative studies with focus on leadership and AMS were included. The literature search was conducted in June 2018 and sorted by best match. We then added articles, which were either retrieved from reference lists or were recommended by experts during various discussions.

### Study setting/design

The Swiss national ethics committee (Project-ID 2018-00361) approved this single center cohort study. All data used for this study were anonymized and the Swiss national ethics committee gave an assistant consent to use the necessary data. Inclusion criteria: All neonates and pediatric patients hospitalized at the NICU/PICU at the Children's Hospital Lucerne between January 1, 2015 and December 31, 2017. On admission, the Clinical Risk Index for Babies II [CRIB II, (23)] was used for preterm infants < 32 weeks of gestational age. The pediatric index of mortality II [PIM II (24)] was used for preterm infants >32 weeks of gestational age and children up to 15 years of age.

The Children's Hospital Lucerne is a teaching hospital for Pediatrics, Pediatric Surgery, and Neonatology. The NICU is a referral level III unit (perinatal center) for central Switzerland covering around 7000-8000 annual deliveries. The PICU cares for children until 16 years of age with health issues from all specialties except cardiac surgery. The NICU/PICU has an accreditation for 11 intensive care beds with about 550–600 admissions per year. The unit is part of the national quality circle for neonatology and pediatric intensive care and provides the requested data of all admissions according to Swiss regulations. Patient's care in the NICU/PICU is provided by one team, consisting of board-certified consultants for neonatology and/or pediatric intensive care, fellows in training for neonatology and/or pediatric intensive care and registrars following a 6-month rotation while in training for general pediatrics. The unit is led by a head consultant who reports to the head of the department of pediatrics. Role descriptions: The head consultant has the clinical and organizational lead of the unit and the ultimate responsibility regarding patient management and outcomes. He is the supervisor of all consultants. Consultants are clinical supervisors of fellows and registrars: They have the daily clinical lead of patient management and are actively involved in regular rounds and prescription of medications. Fellows and registrars conduct the regular rounds, are responsible for patient admission, management, and prescriptions.

### Leadership style

End of August 2015, the clinical and organizational lead of the unit changed. The former head left the hospital and the previous deputy head took over. The rest of the physician team of the unit remained mainly unchanged. Thereby leadership style changed from control-driven to empowering leadership. Obviously, no leader works just with one style, but the contrast of control-driven versus empowering describes best the difference of the two styles. Whereas there is no intent to compare the impact of the diverse leadership styles, there is a possibility to describe the impact of an empowering leadership style due to the distinct change end of 2015.

The focus of leadership after the change was to support and empower frontline physicians on the unit. Within a rough guideline, physicians were asked to treat patients according to their best knowledge within an inter-professional team at the current moment. For example, at the end of 2015 the team came to the agreement to employ an early extubation policy with the goal of extubating patients as soon as possible, rather than waiting to reach specific thresholds or senior approval to do so. No additional request was asked. If doubtful, the physician on-duty always had the possibility to ask senior colleagues for advice. Adverse outcomes such as reintubation within 24 h after extubation were used as a possibility to learn. Another example for a difference within antimicrobial stewardship was related to antibiotic treatment: The rough guideline requested to start antibiotic therapy early in patients with suspected infections. Prescription of the specific antibiotic drug and dose was advised according to a concise, web-based internal guideline. On the other hand, physicians were empowered to stop antibiotic treatment as soon as a bacterial infection was considered to be unlikely, in order to shorten therapy duration as much as possible.

### Antimicrobial stewardship

The NICU/PICU surveillance program was initiated in September 2014 with the goal of collecting prospective data on antibiotic use (general antibiotic days and specifically days with use of meropenem and vancomycin), ventilator associated pneumonia (VAP), and central-line associated blood stream infection (CLABSI). Daily records of patients, ventilation days, suspected VAP, suspected CLABSI and number of patients on antibiotic therapy were obtained by the physician on-duty. The NICU/PICU data manager verified the collected information and fed them regularly into the electronic database.

Since 2007, physicians at the Children's Hospital Lucerne prescribed medications (i.e., antibiotics) according to a web-based internal guideline, which provides advice for correct use and calculates weight-adapted medication dosage. These guidelines were evaluated and adapted every year and since 2016 correspond to the guidelines for infection control of the University Berne (Switzerland) (25). Since 2012, the decision to stop antibiotic therapy in late preterm and term babies with suspected early-onset sepsis was procalcitonin-guided, as the unit was part of the Neonatal Procalcitonin Intervention Study NeoPInS (26). Since the replacement of the units' leadership, multifaceted changes followed: In December 2015, weekly antimicrobial stewardship rounds were introduced with the adult infectious disease specialist. From June 2016 a pediatric infectious diseases specialist consulted the AMS rounds. The “early extubation policy” as described above was initiated in order to shorten duration of invasive ventilation. In January 2016, a VAP-working group was established and their elaborated care bundle was implemented in December 2016. The recommended rules were described by Goerens et al. (27) as the following: “Hand hygiene before and after patient contact and handling respiratory equipment, wearing gloves when in contact with secretions, ventilator circuit changes every 14 days or when visibly soiled, oral care every 2-4 hours, head of bed elevation, draining ventilator condensate before repositioning of the patient, using endotracheal tube (ETT) with cuff, choosing size of the ETT carefully to reduce numbers of reintubation.”

### Definitions

#### VAP

The unit's surveillance program defined suspected VAP according to the following criteria: duration of ventilation of at least 48 h and requested new start or change of antibiotic due to worsening of ventilation conditions and/or clinical deterioration and/or radiological changes compatible with pneumonia and/or changes of tracheal secretions and/or abnormal laboratory parameters (CRP > 20 mg/l, leukocytosis/-penia, I:T ratio >0.2). Proven VAP was defined according to the Center of Disease (CDC) definition (28). In many NICUs, VAP incidence and concomitant antibiotic use were not routinely assessed (29). Nevertheless, suspected pneumonia and VAP is one of the main reasons for an empiric antibiotic therapy in NICUs and therefore we included the neonatal population for the assessment and evaluation of VAP (8). This is in line with recent literature asking to increase neonatologists' interest for VAP (29). Ventilation days were defined as days with invasive ventilation.

#### CLABSI

Suspected CLABSI was defined according to the following criteria: central catheter in place for at least 48 h and new start or change of antibiotic therapy necessary due to worsening of clinical state and/or abnormal laboratory parameters (CRP > 20 mg/l, leukocytosis/-penia, I:T ratio >0.2). Proven CLABSI was defined according to the CDC definitions (30). Catheter days were defined as days with central-line in place.

#### Sepsis/meningitis

Culture proven sepsis and/or meningitis were defined as patients with blood and/or cerebrospinal fluid positive cultures. We excluded patients if blood and/or cerebrospinal fluid culture isolates were considered to be contaminants and the clinical and/or laboratory course was inadequate for sepsis or meningitis. Culture proven infections within the first 48 h of hospitalization were analyzed as community acquired, after 48 h of hospitalization as hospital acquired infections.

#### Correct initial use and streamlining of antimicrobials

In order to assess the use of antibiotics at the unit we evaluated retrospectively empiric choice and streamlining. Streamlining was defined as the first time of narrowing the antibiotic spectrum after receiving results (i.e., Gram stain, identification of germ with resistance pattern) from the microbiology laboratory. Empiric choice of antimicrobial agent was defined according to the web-based internal guideline of the hospital, which is described above. For analysis, correct initial use was defined as an antibiotic treatment that is either the perfect choice or an acceptable choice of medication depending on the case. Likewise, correct streamlining included cases with perfect streamlining, cases in which streamlining was done but more than 24 h after getting the results from the microbiology register and cases, where streamlining was not required. The pediatric infectious disease specialists of the Children's Hospital in Lucerne (MB) and of the Children's Hospital in Zurich (CB) evaluated streamlining and correct initial antibiotic use according to a 4-point Likert scale (perfect, acceptable, not done, not applicable).

### Outcomes

To ensure the comparability of the 3 years (2015, 2016, and 2017) we aimed to compare the following baseline characteristics: CRIB II, PIM II, number of admissions, number of patients, number of preterm infants, ventilation days, catheter days and rate of proven infections (early-onset: within 48 h of hospitalization; late-onset: after 48 h of hospitalization).

The primary outcome was the annual comparison of overall antibiotic days per 1000 patient days and antibiotic days for culture-negative situations (suspected infections and prophylaxis) per 1000 patient days.

The secondary outcomes were defined as: (i) specific antibiotic days of meropenem and vancomycin per 1000 patient days; (ii) the annual comparison of hospital-acquired infections (suspected and confirmed VAP and CLABSI); (iii) antibiotic days for hospital-acquired infections; (iv) antibiotic days for community acquired and hospital acquired culture-proven infections (sepsis and/or meningitis); and (v) the annual comparison of correct initial antibiotic use and correct streamlining for culture-proven infections. We focused on meropenem and vancomycin because both antibiotics are determined as reserve medications within the hospital. Nevertheless, prior to leadership change in September 2015, meropenem was used for treatment of severe abdominal infections, vancomycin for suspected CLABSI.

### Data sources

For analysis of patients' baseline characteristics, data were retrieved from the Minimal Data Set inquiries (MDSi), which is the mandatory data set by the Swiss Society for Intensive Care Medicine (31). The MDSi collects information about number of admissions, number of patients, patient characteristics as age and weight, hospitalization days, PIM II and CRIB II scores, ventilation days and catheter days, and mortality rates. Data collected with the NICU/PICU surveillance program gives information about the number of suspected VAP and CLABSI, such as general antibiotic days, as well as meropenem and vancomycin days. The microbiology register of the Hospital of Lucerne provided the number of positive blood and/or cerebrospinal fluid cultures with discrimination of early (< 48 h after hospitalization) versus late (>48 h after hospitalization) onset sepsis. Details about antibiotic treatment, i.e., kind of antibiotic, length of therapy, date, and time of start of treatment and streamlining, were extracted from patient files and reports of hospitalization.

### Statistical analysis

The rate of observed patient days by the NICU/PICU surveillance program was compared with the number of true patient days retrieved from the MDSi. All results were calculated for 1000 patient days, ventilation days, or catheter days, respectively. Antibiotic days retrieved from the NICU/PICU surveillance program (i.e., antibiotic days overall, meropenem, and vancomycin days) were analyzed on the base of observed patient days by the surveillance program. Antibiotic days retrieved from the microbiology register and from patient files (i.e., antibiotic days for culture-proven infections) were analyzed on the base of true patient days according to the MDSi.

The parameters obtained during antimicrobial stewardship, especially the outcomes mentioned in section 2.6, were analyzed descriptively. In order to assess trends for antibiotic use, e.g., in terms of antibiotic days per 1000 patient days or similarly structured metrics, Cuzick's nonparametric test for trend (32) has been used. Fisher's exact test has been applied to investigate potential associations in contingency tables with binary outcomes. Trends and effects for the incidence of certain events have been evaluated utilizing Poisson regression (adjusted for number of patient days, ventilation days or catheter days where appropriate). Due to the exploratory nature of this retrospective analysis, a significance level of alpha = 5% has been applied without adjustment for multiplicity. However, trends and effects characterized by distinct patterns in the point estimates, supported by *p*-values of *p* < 0.01 or even *p* < 0.001, are considered more likely to be robust.

## Results

### Literature review

**Figure 1:**
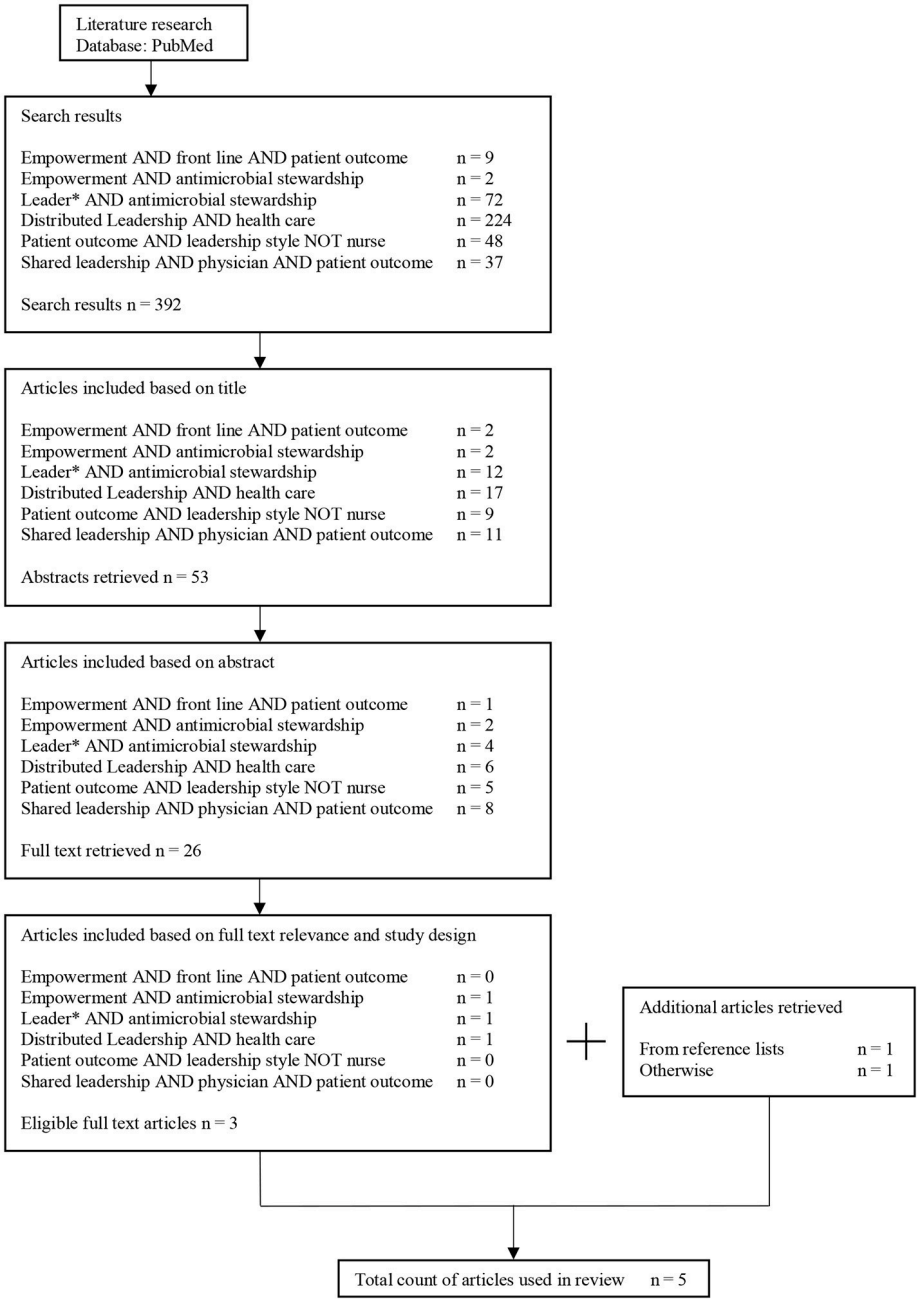
Flow chart illustrating selection process of literature review.

Five articles were included in the review. Figure 1 illustrates the selection process. An overview of the publications is shown in Table 1. Most articles have been published within the last decade, the oldest publication used in the review was published in 2006. Three out of the five articles were qualitative studies. Three studies encompassing leadership and AMS reported patient outcomes. All five studies are in line with the conclusion that an empowering leadership style in one way or another does lead to a higher engagement of staff and/or stakeholders. To share or distribute responsibilities on every team member, it is important to build personal relationships within a team (34) and to establish a penalty free learning culture (37). Positive impacts were reported on culture changes and on AMS topics such as MRSA prevention (35), reduction of nosocomial infections (37) and CLABSI (36).

**Table 1:** Overview of publications used in the review.

**Author**	**Year**	**Study design**	**Setting**	**Title**	**Key messages**	**Main outcome**
Van Buul Laura et al. (33)	2014	Qualitative Study	Tertiary care center, community hospitals, nursing homes and residential care facilities in Netherland	“Participatory action research in antimicrobial stewardship: a novel approach to improving antimicrobial prescribing in hospitals and long-term care facilities”	The study indicates, that the collaborative nature of the participatory action research results in greater engagement compared with top-down approaches	Improvement in antimicrobial prescription
Jeffs et al. (34)	2015	Qualitative study	Intensive care units of 3 teaching hospitals in Toronto and Ontario	“A qualitative analysis of implementation of antimicrobial stewardship at 3 academic hospitals: Understanding the key influences on success”	Successful implementation of an antimicrobial stewardship program should include the following key themes: 1.Get the right people on board; 2.Build collegial relationships (formally and informally) with prescribers; 3.Establishing a track record	Successful implementation of an antimicrobial stewardship program
Sinkowitz-Cochran (35)	2012	Descriptive survey based study	Medical, surgical and intensive care units of Veterans Affairs Medical Centres in the USA	“The associations between organizational culture and knowledge, attitudes, and practices in a multicentre Veterans Affairs quality improvement initiative to prevent methicillin-resistant Staphylococcus aureus”	Greater engagement of healthcare personnel is associated with having a good leadership structure and the feeling of being supported by leadership is positively associated with better MRSA prevention practices	Improvement in MRSA prevention practices leads to reduced infection rate
Pronovost et al. (36)	2016	Quantitative intervention study	Intensive care units in Michigan, USA	“Sustaining Reductions in central line-associated bloodstream infections in Michigan intensive care units: a 10-year analysis”	In order to implement and sustain an improvement in antimicrobial stewardship the active involvement of hospital leaders is important	Reduction of central line-associated bloodstream infections
Jain et al. (37)	2006	Quantitative intervention study	Single intensive care unit in Mississippi, USA	“Decline in ICU adverse events, nosocomial infections and cost through a quality improvement initiative focusing on teamwork and culture change”	Adverse events and nosocomial infections declined following the introduction of a changed system of care in the ICU	Reduction of nosocomial infections after intervention

### Study population

During the study period (January 2015 to December 2017), a total of 1567 patients were admitted to the NICU/PICU and included in the study population. Baseline characteristics are listed in Table 2. 6722 out of 7875 (85%) patient days were observed within the PICU/NICU surveillance program. We observed a downward trend of the annual number of ventilation days and catheter days from 2015 to 2017 (*p* < 0.001). Mortality over the study period showed a not significant, decreasing trend from 2.1% in 2015 to 1.5% in 2017 (Table 3).

**Table 2:** Baseline characteristics of study population on admission.

	**2015**	**2016**	**2017**
**ADMISSIONS AND PATIENTS**			
Admissions (*n*)	556	575	571
Patients total (*n*)	521	518	528
Newborns < 44 weeks of gestational age (n) Percentage of newborns < 44 weeks on gestational age on patients total (%)	302 58.0	299 57.7	291 55.1
Preterm infants < 32 weeks of gestational age (*n*) Percentage of preterm infants < 32 weeks of gestational age on patients total (%)	63 12.1	76 14.7	82 15.5
Preterm infants < 28 weeks of gestational age (*n*) Percentage of preterm infants < 28 weeks of gestational age on patients total (%)	22 4.2	23 4.4	26 4.9
**ADMISSION SCORES**			
CRIB II (mean ± SD)	6.5 (±2.8)	6.0 (±2.8)	5.7 (±2.5)
PIMS II (mean ± SD)	3.4 (±9.7)	3.9 (±9.9)	5.4 (±12.3)

**Table 3:** Annual comparison of outcomes.

	**2015**	**2016**	**2017**	***P* values**
**ANTIBIOTIC USE OVERALL**				
Patient days (*n*)	2628	2729	2518	NA
Antibiotic days per 1000 patient days (*n*)	474.1	398.3	403.9	*p* < 0.001^a^
Antibiotic days for culture-negative situations per 1000 patient days (*n*)	418.2	358.0	309.4	*p* < 0.001^a^
Meropenem days per 1000 patient days (*n*)	53.2	27.4	25.5	*p* < 0.001^a^
Vancomycin days per 1000 patient days (*n*)	86.8	43.1	33.1	*p* < 0.001^a^
**HOSPITAL ACQUIRED INFECTIONS: VAP**				
Ventilation days (*n*) Percentage of ventilation days on patient days (%)	956 36.4	739 27.1	545 21.6	*p <* 0.001^a^
Suspected VAP per 1000 ventilation days (*n*)	26.2	19.0	9.2	*p* = 0.027^c^
Proven VAP per 1000 ventilation days (*n*)	3.1	0	0	NA
Antibiotic days for suspected VAP per 1000 ventilation days (*n*)	214.4	150.2	56.9	*p <* 0.001^a^
Antibiotic days for proven VAP per 1000 ventilation days (*n*)	32.4	0	0	p < 0.001^a^
**HOSPITAL ACQUIRED INFECTIONS: CLABSI**				
Catheter days (*n*) Percentage of catheter days on patient days (%)	1687 64.2	1618 59.3	1197 47.5	*p <* 0.001^a^
Suspected CLABSI per 1000 catheter days (*n*)	10.7	8.7	6.7	*p* = 0.261^c^
Proven CLABSI per 1000 catheter days (*n*)	1.8	0.6	0	*p* = 0.156^c^
Antibiotic days for suspected CLABSI per 1000 catheter days (*n*)	70.0	63.7	33.4	*p <* 0.001^a^
Antibiotic days for proven CLABSI per 1000 catheter days (*n*)	17.8	4.3	0	*p <* 0.001^a^
**CULTURE PROVEN INFECTIONS**				
Positive blood cultures (*n*)	10	10	17	*p* = 0.129^d^
Positive blood cultures within 48 h of hospitalization (= community acquired infections)	4	2	12	*p* = 0.034^b^
Positive blood cultures after 48 h of hospitalization (= hospital acquired infections)	6	8	5	
Positive CSF cultures (*n*)	1	0	0	NA
Antibiotic days for culture proven infections per 1000 patient days (*n*)	55.9	40.3	94.5	*p <* 0.001^a^
Antibiotic days for community acquired, culture proven infections per 1000 patient days (*n*)	14.8	8.4	71.5	*p <* 0.001^a^
Antibiotic days for hospital acquired, culture proven infections per 1000 patient days (*n*)	41.1	31.9	23.0	*p <* 0.001^a^
Percentage of antibiotic days for culture proven infections (%)	11.8	10.1	23.4	NA
Correct initial antibiotic use (*n*) Percentage of correct initial antibiotic use (%)	9/10 90.0	9/10 90.0	14/17 82.4	*p* = 1^b^
Correct streamlining (*n*) Correct streamlining (%)	7/10 70.0	8/10 80.0	12/17 70.6	*p* = 0.902^b^
**MORTALITY**				
Mortality all patients (*n*) Mortality all patients (%)	11 2.1	8 1.5	8 1.5	*p* = 0.760^b^
Mortality newborns < 44 weeks of gestational age (*n*) Mortality newborns < 44 weeks of gestational age (%)	10 3.3	6 2.0	5 1.7	*p* = 0.467^b^

a *Cuzick's nonparametric test for trend*;

b *Fisher's exact test*;

c *Poisson regression (adjusted for number of ventilation or catheter days)*;

d *Poisson regression (adjusted for number of patient days); NA, not applicable*.

### Primary outcomes

Annual antibiotic days per 1000 patient days declined significantly from 474.1 in 2015, to 398.3 and 403.9 days in 2016 and 2017, respectively (*p* < 0.001). Antibiotic days for culture negative situations (suspected infections and prophylaxis) decreased significantly from 418.2 in 2015, to 358.0 and 309.4 days per 1000 patient days in 2016 and 2017, respectively (*p* < 0.001) (Table 3).

### Secondary outcomes

The use of meropenem and vancomycin decreased significantly (*p* < 0.001) over the 3 years: Meropenem days per 1000 patient days decreased from 53.2 in 2015, to 27.4 and 25.5 in 2016 and 2017, respectively; Vancomycin days from 86.8 in 2015, to 43.1 in 2016 and 33.1 in 2017.

During the 3 years we noted a decreasing tendency of suspected and proven hospital-acquired infections: The rate of suspected VAP-episodes decreased significantly from 26.2 per 1000 ventilation days in 2015 to 19.0 in 2016 and 9.2 in 2017 (*p* = 0.027). In 2015, 3.1 proven VAP-episodes per 1000 ventilation days were noted, whereas none in 2016 and 2017. The rate of suspected CLABSI-episodes decreased not significantly from 10.7 per 1000 catheter days in 2015 to 8.7 in 2016 and 6.7 in 2017 (*p* = 0.261). In 2015, 1.8 proven CLABSI-episodes per 1000 catheter days were noted, 0.6 in 2016 and none in 2017.

Antibiotic days per 1000 ventilation days for suspected VAP declined from 214.4 in 2015, to 150.2 in 2016 and 56.9 in 2017 (*p* < 0.001). For proven VAP, in 2015 32.4 antibiotic days per 1000 ventilation days were noted, whereas none in 2016 and 2017 (*p* < 0.001). Antibiotic days per 1000 catheter days for suspected CLABSI declined from 70.0 in 2015 to 63.7 in 2016 and 33.4 in 2017 (*p* < 0.001). For proven CLABSI, 17.8 antibiotic days per 1000 catheter days were noted in 2015, 4.3 in 2016 and none in 2017 (*p* < 0.001).

The percentage of antibiotic days used for culture-proven infections (sepsis and/or meningitis) increased from 11.8% and 10.1% in 2015 and 2016 to 23.4% in 2017. The number of culture-proven infections and concomitant antibiotic days increased significantly (*p* < 0.001) due to an increase of community acquired infections. The number of hospital acquired, culture-proven infections, and concomitant antibiotic days decreased significantly (*p* < 0.001). The annual comparison of correct initial antibiotic use for culture proven infections and the rate of correct streamlining remained mainly unchanged. The rate of correct initial antibiotic use for culture proven infections was between 82 and 90%, streamlining was correct in 80–90% of the cases.

**Figure 2:**
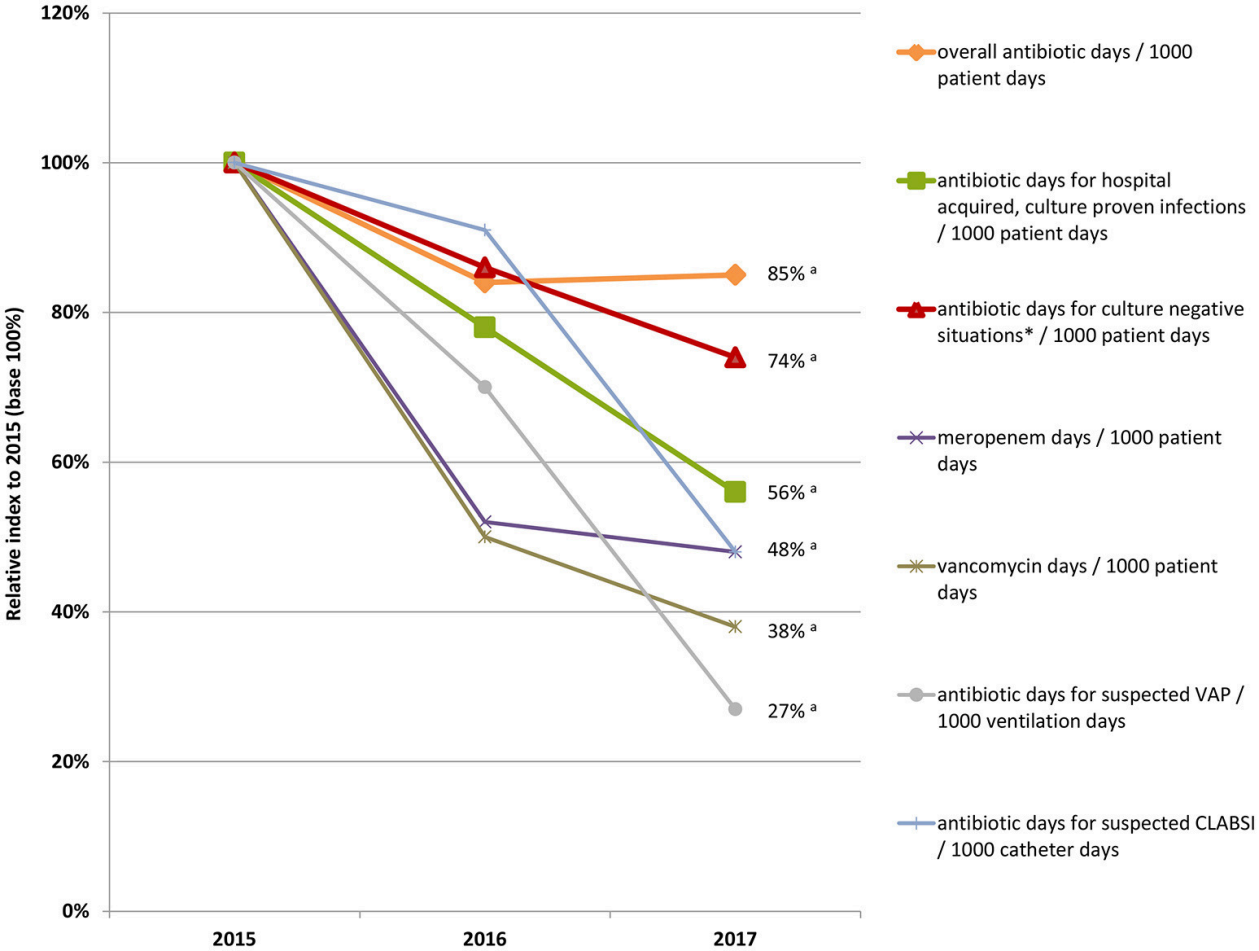
Relative annual index to 2015 (base 100%) of antibiotic days overall, meropenem and vancomycin days, and antibiotic days for hospital acquired, culture-proven infections, and culture-negative situations per 1000 patient days; not shown are antibiotic days for community acquired, culture-proven infections because development of infection is independent of the unit; ^*^Culture-negative situations: suspected infections and prophylaxis; ^a^Cuzick's non-parametric test for trend: *p* < 0.001.

Table 3 gives an overview of all results. Figure 2 depicts the results as relative annual index to 2015 (base 100%).

## Discussion

The annual comparison of overall antibiotic days per 1000 patient days and antibiotic days for culture-negative situations showed a significant improvement over the 3 years. Furthermore, hospital-acquired infections such as VAP and CLABSI decreased and the use of broad-spectrum antibiotics such as meropenem and vancomycin was reduced by more than 50%. Meanwhile, mortality rates and culture-proven, hospital-acquired infections remained unchanged. These findings indicate that quality of antibiotic use and infection control improved significantly. Correct initial antibiotic use was around 80–90% and streamlining remained unchanged by 70–80% over the study period.

Antibiotic days per 1000 hospitalization days are difficult to compare between different units as NICU and PICU's often have different infrastructures and diverse patient spectra. Therefore, it is not possible to assess the absolute number of 400 antibiotic days per 1000 patient days. Similarly, the comparison of meropenem and vancomycin use within the literature is difficult to assess. However, there are a few reports in line with our findings with successful reduction of their use in time series in NICU/PICUs after introducing an AMS (38–40). Analog, there are reports regarding a high variation of antibiotic use in different NICUs with unchanged outcomes indicating potential overuse (5, 41, 42). Cantey reports a rate of 89% of empirically prescribed antibiotics for culture-negative situations in their NICU which is in line with our findings in the years 2015 and 2016 (9). With increasing numbers of multi-resistant bacteria particularly on ICU's and the growing body of knowledge on the negative effects of antibiotics on the individual microbiome with potential impact on future health, there is a mandatory request for every unit to assess, evaluate and minimize antibiotic use (11, 13–19). The structural interventions which were introduced to our unit had a clearly positive effect on overall antibiotic use, reducing the overall consumption and increasing the rate of antibiotics used for culture-proven infections. We do interpret this as a step in the aimed direction. Furthermore, mortality rates within the study period showed a decreasing trend, which was not statistically significant due to the low rate of mortality. Nevertheless, recent publications underline this result, showing an association between overuse of antibiotics and increased short-term morbidity and mortality (13).

With successful implementation of AMS programs it is possible to reduce hospital-acquired infections as VAP and CLABSI rates (36, 37, 43). The observed rate of 3.1 VAP episodes per 1000 ventilation days and 1.8 CLABSI episodes per 1000 catheter days in 2015 is within the published range of hospital-acquired infections (29, 44). Nevertheless, in 2017 we achieved a blank sheet without any confirmed VAP or CLABSI. Most studies report only rates of confirmed hospital-acquired infections as VAP and CLABSI, which has become accepted quality indicators for intensive care units. Our findings indicate that there is a high variance between suspected and proven hospital-acquired infections with concomitant antibiotic use. Fisher et al. reported that 47% of antibiotic prescriptions in a PICU were due to suspected VAP (8). Cantey reported that 62% of antibiotic courses over 5 days for culture-negative situations in their NICU were for suspected pneumonia (9). Therefore, assessment, evaluation, and reduction of suspected and confirmed hospital-acquired infections has to become the standard measure for quality improvement initiatives.

In this single center study, AMS in regards to antibiotic use and hospital-acquired infections improved remarkably over the 3 years. The unit's surveillance program for AMS was introduced in October 2014 and therefore already in place 2015. The new head of unit did not just set up an AMS program because he had the goal to do so. Through multifaceted changes after discussion within the team, i.e., the early extubation policy, team members were empowered to take over direct responsibility for their patients. Based on the overarching goal to optimize care, AMS was just part of it and goals were set through everyone on the team. This is a key message of successful change management: The statement “successful change leadership involves investing time in finding common ground across stakeholders and in building credibility and trust“ (45) accentuates the fact, that in order to successfully implement a change, stakeholders need to define their own goals (46, 47). The potential of empowerment as leadership style lies in the fact that the members of the team receive a sense of meaning and coherence through their self-developed goals. This also leads to the phenomenon that the team members remain committed to the project because they want to achieve their previously self-defined visions. The leader does not delegate orders top-down, but builds a team of direct caregivers and empowers them to take over responsibility and make their own decisions. The leader is perceived as a coach (48). Leadership who enables team members to take over direct responsibility and which emphasize the importance of a positive team atmosphere is well known in industries outside health care (49, 50). Literature in health care is relatively scarce and mainly focused on nursing staff (38). Those articles often focus only on the qualitative impact of leadership style on the team such as job satisfaction (51, 52).

The need of a strong leadership in order to implement a quality improvement program is mentioned in several studies (46, 53, 54). The CDC guidelines for the implementation of an AMS program mention leadership commitment as the first of their 7 core elements (55). The wording “strong leadership” though bears the potential of different definitions of leadership styles. Obviously, a rather directive, control-driven leadership style can be strong and effective: In trauma care the optimal style and leadership depends on patient characteristics and team composition. Directive leadership in an emergency trauma room is most effective when pressure and urgency is high and teams are inexperienced (45). But individual competence and autonomy was a corner stone of physician's education and development during the last few decades (56–58). Therefore, physicians often do not like to be told what to do and prescriber opposition is one of the barriers of successful implementation of AMS programs (21, 59). This is in contrast to the current recommendations of AMS programs: The strategy with the best evidence consists of a multidisciplinary team, which ideally includes an infectious diseases physician (leading/directing the program), a clinical pharmacist with infectious diseases training, a clinical microbiologist, an information system specialist, an infection control professional and a hospital epidemiologist (2). The AMS team is like a control system giving directive orders to the frontline physicians. Empowering leadership for front-line physicians regarding AMS is an alternative way and may improve implementation of AMS even when the respective staff and financial resources for a comprehensive AMS program are not available.

The few articles that we found within the literature review regarding leadership and AMS agree that an empowering leadership style leads to a higher engagement of staff and may have positive impacts on prevention of MRSA infections (35), reduction of nosocomial infections (37) and CLABSI (36). This is in line with the results of our study that the significantly reduced rate of hospital-acquired infections is at least partly due to the empowering leadership style. As example, the fact that physicians as direct caregivers were enabled to extubate earlier leads to shorter duration of ventilation, reduces the risk of VAP, and may be collaterally responsible for the reduction of catheter days with a reduced risk of CLABSI. Similar, to stop antibiotic therapy early in culture negative situations may be seen as evident, best practice. Nevertheless, frontline staff needs to be empowered to do so through education and without being at risk to be blamed. The prerequisite for empowering team members is education (37): Staff members need to be educated before and while an intervention or change, in order to gain the required knowledge and skills to be able to take over responsibilities. Therefore, an empowering leadership style requires not only a compliant leader, but also a suitable team. Jeffs et al. describe it as “getting the right people on board” (34). Jain et al. mentioned a culture change with better communication within a multidisciplinary team and a stronger feeling of penalty-free unity as an important strategy in order to reduce nosocomial infections and having a better coordinated team (37). On the other hand, actions like the stewardship rounds held once a week with the infectious disease specialist in our unit seem to be insufficient to improve streamlining. Similar, the practice of correct initial use of antibiotics was high with 90% in the first 2 years of the study, but showed a decreasing trend. Whereas streamlining is probably less influenced by empowering leadership and more a problem of knowledge and education, reduced correct initial antibiotic therapy may be a side effect of empowering and individual autonomy of physicians.

Our study has several limitations. Obviously and most important, the connection between the significantly improved AMS data and the change of leadership style is only an association. There were multifaceted changes during the study period and therefore there is no proof of this relationship. Through leadership change and multifaceted changes the awareness and compliance for infection precautions and rational antibiotic use may increase. On the other hand, many parts of the implemented changes as for example early extubation or early stop of antibiotic therapy were dependent on empowered frontline physicians encouraged to do it. Obviously, it is possible that similar results could be achieved through daily visits of an infectious disease specialist and by strengthening the multidisciplinary approach. However, empowering leadership may serve as an add on or an option if resources are limited. Furthermore, the analysis uses the outcome data of 2015 as baseline and we have no data to show that they are representative for former years. Third, we did not measure the change of leadership style objectively. Nevertheless, we observed a significant improvement over a period of 3 years with a mainly unchanged physician team and steady baseline characteristics of admitted patients.

## Conclusion

Based on our findings with clearly improved antibiotic use and reduced rate of hospital-acquired infections, we conclude that an empowering leadership style which focuses on enabling frontline physicians to take over direct responsibilities for their patients may be a successful strategy to improve antimicrobial stewardship.

## Author contributions

The project was devised by MS, data collection and writing of the manuscript including the literature review was done by KES, closely revised by MS. Statistical calculations were done by DL. Collection of data based on the NICU/PICU surveillance program was done by KS, KD, MF, DM, KG, KO, PG, SB, PS, UT, and MS. Critical revising of the work was done by CB, MB, KS, KD, MF, DM, KG, KO, PG, SB, PS, and UT. All authors read and approved the submitted version of this manuscript.

### Conflict of interest statement

The authors declare that the research was conducted in the absence of any commercial or financial relationships that could be construed as a potential conflict of interest.

## Abbreviations

AMS: Antimicrobial stewardship
NICU: Neonatal intensive care unit
PICU: Pediatric intensive care unit
VAP: Ventilator associated pneumonia
CLABSI: Central line associated blood stream infection
MDSi: National minimal data set intensive care
EOS: Early onset sepsis
LOS: Late onset sepsis
CRIB II: Clinical risk index for babies II
PIM II: Pediatric index of mortality II
SAPS II: Simplified acute physiology Score II
CDC: Center for Diseases Control and Prevention
NEC: Necrotizing enterocolitis
ETT: Endotracheal tube
I:T ratio: Immature neutrophils to total neutrophils
CRP: C-reactive protein
HAI: Hospital-acquired infection.
